# Supplementary material for: The influence of glottal and respiratory factors on aerosol emission during phonation
Source: PLoS One. 2025 Dec 5;20(12):e0321909. doi: 10.1371/journal.pone.0321909 (PMC12680239; doi:10.1371/journal.pone.0321909)
Supplement: Attachment — Submitted filename: report_10.1101+2025.03.16.25324059.pdf [file pone.0321909.s001.pdf]

ScreenIT Report

This is an automated report for 10.1101+2025.03.16.25324059.pdf. This report was solicited by the PLOS One editorial team and provided by ScreenIT.

ScreenIT is an independent group of scientists developing automated tools that analyze academic papers. A set of automated tools screened your submitted manuscript and provided the report below. Each tool was created by your academic colleagues with the goal of helping authors.

The tools look for factors that are important for transparency, rigor and reproducibility, and we hope that the report might help you to improve reporting in your manuscript. Within the report you will find links to more information about the items that the tools check. These links include helpful papers, websites, or videos that explain why the item is important.

While our screening tools aim to improve and maintain quality standards they may, on occasion, miss nuances specific to your study type or flag something incorrectly. Each tool has limitations that are described on the ScreenIT website. The tools screen the main file for the paper; they are not able to screen supplements stored in separate files.

Please note that the Academic Editor had access to these comments while making a decision on your manuscript. The Academic Editor may ask that issues flagged in this report be addressed.

If you would like to provide feedback on the ScreenIT tool, please email the team at ScreenIt@bih-charite.de. If you have questions or concerns about the review process, please contact the PLOS One office at plosone@plos.org.

How to read the report:

- no action required
- review for potential issues
- a critical issue was found by a tool
- may not be applicable to your paper

- more information about this item can be obtained using this link

Rigor

✓	Ethics	IRB: *"The study was approved by the University of Colorado Boulder Institutional Review Board (IRB Protocol #200282) for the aerosol measurements and the Colorado Multiple Institutional Review Board (COMIRB Protocol #22-1678) for the laryngoscopy."* Consent: *"Participants were provided with informed written consent."*
?	Inclusion and Exclusion Criteria	Not detected. Please specify any pre-defined criteria used to determine whether participants, animals or samples were eligible for the study, or exclude participants, animals or samples from the study.
?	Flow charts and attrition	We did not find a study flow chart of excluded observations. We strongly recommend using flow charts because they provide an overview of the study design and more information about attrition. If you included a study flow chart in your supplemental files, we apologize for missing this. Our tool is not able to screen separate supplemental files.<br><br>Sentence about attrition: *"9 Methods Participants This experiment included five healthy female participants, all between the ages of 22 43 years (mean = 31), with no history of voice or respiratory impairments."*
✓	Sex as a biological variable	*"9 Methods Participants This experiment included five healthy female participants, all between the ages of 22 43 years (mean = 31), with no history of voice or respiratory impairments."*
?	Subject Demographics	Age: not detected.<br>Weight: not detected.
?	Randomization	Not detected. If you performed an experimental study, please specify whether participants, animals or samples were randomly assigned to treatments or groups, and specify the randomization procedure.

?	Blinding	Not detected. Please specify whether blinding was used at various phases of the experiment (e.g., blinding of patients, caregivers, outcome assessments, data analysis).
?	Power Analysis	Not detected. Please state how you determined the sample size for the study. If you performed a power or sample size calculation, please provide details.
n/a	Replication	not required.

Generated by Barzooka (flow charts), SciScore (everything else).
SciScore only screens the text of the methods section; we apologize if the tool has missed items reported elsewhere in the paper or supplements.

Resources

Persistent unique identifiers for research resources, RRIDs, can be used to track these key resources though the literature. They are requested or required in many journals, but even if your journal does not require them, we strongly encourage the use of these for the readers of papers who wish to easily track down the key materials like antibodies, software tools, or mice.

Software and Algorithms	*"The normalized glottal gap was then measured for each still shot using ImageJ (National Institutes of Health, Bethesda, MD, USA, https://imagej.nih.gov/ij) based on the procedures used in (31)."*	? ImageJ suggested: ImageJ, RRID:SCR_003070. Please make sure this RRID suggestion is accurate before including in your manuscript.

Software and Algorithms	*"HNR was computed using Praat software (32) from the audio from the laryngoscopy videos to quantify vocal fold periodicity by participant and phonation task."*	Praat suggested: Praat (RRID:SCR_016564). Please make sure this RRID suggestion is accurate before including in your manuscript.

Generated by SciScore.
SciScore only screens the text of the methods section; we apologize if the tool has missed items reported elsewhere in the paper or supplements.

Transparency

?	Open code	We found one or more references to open code in your manuscript that did not seem to be included in your code availability statement: *"data and code can be found at https://osf.io/uc6km/."*.If these are references to your own code, make sure to include them in the code availability statement. If these are code citations, make sure to clearly state that you used previously published code.
?	Open data	We found one or more references to open data in your manuscript that did not seem to be included in your data availability statement: *"FALSE"*.If these are references to your own data, make sure to include them in the data availability statement. If these are data citations, make sure to clearly state that you used previously published data.

✓	Self-acknowledged limitations	*"While compelling, the results of this study had several limitations. First, the small sample size limits generalizability, and future studies should include larger, more diverse participant groups. Second, only sustained vowel production was used to attempt to isolate aerosol generation at the laryngeal level (and not from movement in the oral cavity). Future studies should examine aerosol production in naturalistic conversational speech, as individuals vary their speech intensity, airflow, and articulation dynamically. For example, projected speech in noisy environments (e.g., classrooms, restaurants) may increase aerosol emissions, while quiet, modal speech in one-on-one settings may minimize risk. Investigating these real-world speech variations would help refine public health guidelines for airborne disease mitigation in different social contexts. Finally, there is evidence that the APS is less efficient at measuring liquid particles 10 m or higher (34), however it is not well unde..."*
?	Conflict of interest statement	It is important to supply a conflict of interest statement, even if there are none to declare, to transparently present the research.
✓	Funding statement	Thank you for reporting your funding source(s), or stating that there was no specific funding for this work.
n/a	Registration statement	A registered protocol makes the planned research project more transparent and can protect against bias. Please consider registering future confirmatory studies.

Generated by ODDPub, limitation-recognizer, rtransparent, trial-identifier.

Figures

?	Bar graphs of continuous data	We found bar graphs of continuous data. We recommend replacing these with more informative graphics (e.g. dotplots, box plots, violin plots), as many different datasets can lead to the same bar graph. The actual data may suggest different conclusions from the summary statistics alone. Please see the following resources for more information on the problems with bar graphs (Weissgerber et al 2015) and what to use instead (Weissgerber et al 2019).

	No rainbow color maps	Thank you for not using rainbow colormaps. Rainbow color maps distort readers’ perception of the data by introducing visual artifacts and are not accessible to colorblind readers.

Generated by JetFighter, Barzooka.
