# Supplementary material for: The influence of glottal and respiratory factors on aerosol emission during phonation
Source: PLoS One. 2025 Dec 5;20(12):e0321909. doi: 10.1371/journal.pone.0321909 (PMC12680239; doi:10.1371/journal.pone.0321909)
Supplement: Attachment — Submitted filename: Reviewer_Feedback.docx [file pone.0321909.s002.docx]

Department of Speech, Language, and	t	303 492 3404

Hearing Sciences	f	303 492 3274

2501 Kittredge Loop Rd.

409 UCB

Boulder, Colorado 80309-0409

October 29, 2025

Steven R Livingstone

Academic Editor

PLOS ONE

**Manuscript title:** *Aerosol Emission During Speech: Investigating the Role of Glottal Configuration and Respiratory Effort*
**Manuscript ID:** PONE-D-25-11745
**Authors:** Hilger et al.

Dear Dr. Livingstone,

We thank the editor and Reviewer One for their thoughtful and constructive feedback. We have revised the manuscript substantially to address all major and minor points. Below, we provide a point-by-point response. Reviewer comments are in **bold**, followed by our responses in regular text. We have made the revisions to this manuscript using red ink.

I want to note that even though Reviewer One said that they do not need to review the paper again after addressing these comments, we would like to extend our gratitude for their time and expertise. It is evident that they spent quite a bit of time reviewing the paper and providing constructive feedback. They were wonderful to work with.

Thank you,

Allison Hilger, Tehya Stockman, Corey Murphey, Jacqueline McCurdy, and Shelly Miller

**Reviewer #2: Dear Authors,**

**This manuscript is MUCH, MUCH improved. Thank you for taking on all of my very, very detailed points! I have one or two tiny little points that still, to my mind, need addressing - but they are minor. Once these are addressed, I think this manuscript needs to be accepted (and I don't need to review it again).**

**Line 46 - Call me a stickler (which I'm sure you already have, lol) - but I'm not sure you can have 'voiceless phonation' since phonation is typically used to refer to vocal fold vibration. Here, and elsewhere, it might be better to call whispering a 'voiceless sound production', or even 'voiceless production'.**

- Response: Great point. This wording has been revised.

**Line 50 - Following on from above, 'sustained sound production'**

- Response: This has been revised.

**Line 137 - 'followed by MODAL PHONATION'**

- Response: This has been revised.

**Line 159 - I only used /^/ since it's what I can produce on my keypad, but please use the real IPA symbol for this: /ʌ/**

- Response: This has been revised.

**DISCUSSION**

**Line 458 - /h/-onset shouldn't have any wider glottal configuration during phonation (unless it encouraged a slightly breathier phonation style, which you weren't aiming at), and whispering doesn't have phonation. Maybe reword to 'wider glottal configurations' without the 'phonation'. I can see the h-onset NGG during the vowel was actually larger than for modal voice, but within the error bar (Figure 4). It's possible that the task did induce some participants to produce a slightly breathier voice quality? Anyway, you can probably make the same point by saying 'Tasks characterised by wider glottal configurations (e.g. whispering, loud phonation)**

- Response: This has been revised to remove reference to phonation. I do agree that this task likely led to a slightly breathier voice quality.

**Figure 4 - Need to change the 'Loud Speech' labels to 'Loud Modal'**

- Response: Loud speech has been revised to Loud Modal in this figure.

**Line 461 - You elicited forced, 'high intensity' (line 162) whispering - so not sure you can really call this a 'quieter production' (especially without even relative SPL values to go on). Also, I'm not familiar with the other literature you cite (11,12) - was their whispering a loud, forced whisper or a whisper one might use to not be overheard. Are your findings really 'contrary to the common assumption that quieter productions carry lower risk'? This needs a bit of rewording**

- Response: Great point. We have revised this section to say, “Although whispering is often perceived as a quieter mode of voice production, the forced, high intensity whispering elicited in our task was associated with high aerosol concentrations. Whisper tasks can involve a wider glottal configuration and substantial ventilatory output, which is consistent with the observed levels; however, mechanisms (e.g., flow or turbulence) were not measured and are not inferred. Thus, our findings highlight that not all forms of whispering should be assumed to entail lower aerosol emission.”

**476-486 - I'm not sure that mentioning 'lung volumes' is necessary. Keep line 478 up until, 'therefore requiring initiation of phonation at a higher lung volume', which you can drop. Then you can remove 482-484. I think you can just say that loud voicing typically requires higher SG pressure and stronger expiratory force AND GREATER AIRFLOWS'. Then in 485, 'Because airflows were not recorded...'**

- Response: Excellent suggestions. This has been revised exactly according to these suggestions.

**498 - 'whispering, a voiceless PRODUCTION task'**

- Response: This has been revised.

**509-517 - This does need some nuancing. As mentioned, not all whispers are equal! Line 510 might be better as, 'whispering, ESPECIALLY NOT A FORCED, STAGE WHISPER, is not necessarily a safer alternative...' You have not measured a 'confidential whisper', so, I'm not sure you can say that a clear, low intensity modal register is likely to generate fewer particles. I would be surprised if a genuine, 'confidential' whisper - produced with a similar airflow to modal voice - did not generate fewer particles than quiet modal voice as it would essentially be the same task without vocal fold contact. That said, since Joe Public probably does not know that there's a difference between a quiet whisper and a loud whisper, I think it does bear reporting that a whisper is not necessarily safe. However, I think this paragraph needs some stronger qualifications and caveats. I know you talk about this in the limitations section (line 539), but it needs an extra nod here.**

- Response: Great points. We have added more nuance to this section:

“These findings have important implications for airborne disease transmission. As also noted in prior work (11,12), whispering is not necessarily a safer alternative for reducing aerosol emissions, particularly when produced as a forced or “stage” whisper, as in our task. Our study did not examine confidential, low intensity whispering, and it remains possible that such productions, with airflow closer to modal voice, may yield lower particle counts. Still, the high concentration of small particles (0.1–1 µm) observed in forced whispering and loud voicing suggests that these tasks may contribute meaningfully to airborne disease spread, as smaller particles remain suspended in the air longer and travel further. In addition, the production of larger particles (10–20 µm) during whispering raises concerns about fomite transmission, since such droplets can settle on surfaces.”

**Line 532 - NGG is anything but stable. It will vary over the course of a single vowel from onset to termination, across repetitions of the same vowel, vary at different pitches and intensities (as you say in 535 - instantaneous glottal area which is NGG). That said, intra-participant variation across tasks types are likely to be large enough that the metric is useful for your purposes. I think this is the point you are making?**

- Response: Great points here as well. We have added more nuance to this section:

“While NGG is known to vary within and across productions (e.g., with onset, repetition, pitch, or intensity), here it is used as a participant-level characteristic to capture general tendencies rather than a task-specific state.”
